# Modular regulation of floral traits by a *PRE1* homolog in *Mimulus verbenaceus*: implications for the role of pleiotropy in floral integration

**DOI:** 10.1093/hr/uhac168

**Published:** 2022-07-27

**Authors:** Hongfei Chen, Zheng Xiao, Baoqing Ding, Pamela K Diggle, Yao-Wu Yuan

**Affiliations:** Department of Ecology and Evolutionary Biology, University of Connecticut, Storrs, CT 06269, USA; Department of Ecology and Evolutionary Biology, University of Connecticut, Storrs, CT 06269, USA; School of Landscape Architecture, Zhejiang A&F University, Hangzhou 311300, China; Department of Ecology and Evolutionary Biology, University of Connecticut, Storrs, CT 06269, USA; College of Horticulture, Nanjing Agricultural University, Nanjing 210095, China; Department of Ecology and Evolutionary Biology, University of Connecticut, Storrs, CT 06269, USA; Department of Ecology and Evolutionary Biology, University of Connecticut, Storrs, CT 06269, USA; Institute for Systems Genomics, University of Connecticut, Storrs, CT 06269, USA

## Abstract

Floral traits often show correlated variation within and among species. For species with fused petals, strong correlations among corolla tube, stamen, and pistil length are particularly prevalent, and these three traits are considered an intra-floral functional module. Pleiotropy has long been implicated in such modular integration of floral traits, but empirical evidence based on actual gene function is scarce. We tested the role of pleiotropy in the expression of intra-floral modularity in the monkeyflower species *Mimulus verbenaceus* by transgenic manipulation of a homolog of *Arabidopsis PRE1*. Downregulation of *MvPRE1* by RNA interference resulted in simultaneous decreases in the lengths of corolla tube, petal lobe, stamen, and pistil, but little change in calyx and leaf lengths or organ width. Overexpression of *MvPRE1* caused increased corolla tube and stamen lengths, with little effect on other floral traits. Our results suggest that genes like *MvPRE1* can indeed regulate multiple floral traits in a functional module but meanwhile have little effect on other modules, and that pleiotropic effects of these genes may have played an important role in the evolution of floral integration and intra-floral modularity.

## Introduction

A flower is essentially a functional unit composed of multiple floral organs that must be arranged in a proper configuration to ensure pollination success. As such, floral traits often show correlated variation within and among species [1–6], a pattern commonly referred to as floral integration [7–9]. For sympetalous species (i.e. species bearing flowers with their petals fused into a corolla tube), strong correlations among corolla tube, stamen, and pistil length are prevalent [10–18]. These three traits likely form a common intra-floral functional module, as their coordination is critical for successful pollen transfer [8]. This raises the question: what is the genetic architecture underlying such intra-floral modules?

Pleiotropy and linkage disequilibrium are two main genetic hypotheses for the expression of modularity [19–24]. The pleiotropy hypothesis assumes that a single gene affects multiple traits simultaneously within a module, whereas the linkage disequilibrium hypothesis assumes that each individual trait within the module is controlled by a different gene but some mechanism (e.g. recombination suppression associated with heterochromatic regions or chromosomal inversions) maintains a tight linkage among these genes. Of the two hypotheses, pleiotropy has been considered a major driver of floral integration and intra-floral modularity [2, 9, 10], although conclusive evidence supporting this hypothesis is scarce. The strongest evidence so far probably comes from studies of wild radish (*Raphanus*) and tobacco (*Nicotiana*) flowers, where genetic correlations among corolla tube, stamen, and pistil length do not break down after several generations of random mating [2, 10]. However, even in these cases the actual gene that presumably affects all three traits has not been identified, and the possibility that these traits are controlled by multiple distinct genes in a chromosomal region with recombination suppression cannot be completely ruled out.

**Figure 1:**
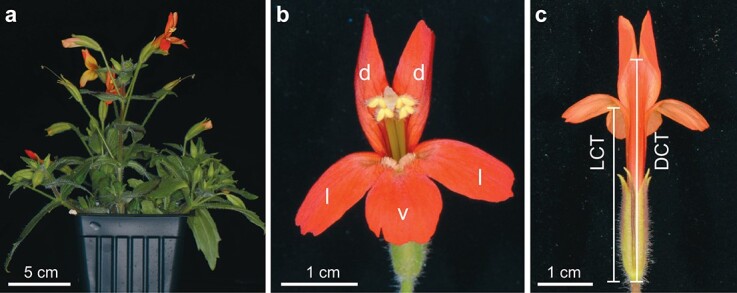
Phenotypes of the *Mimulus verbenaceus* inbred line MvBL. (a) Side view of a 10-week old plant. (b) Face view of the MvBL flower. d: dorsal petal lobe; l: lateral; v: ventral. (c) Back view of the MvBL flower. Lengths of both the dorsal corolla tube (DCT) and the lateral corolla tube (LCT) were measured in this study.

An alternative strategy to test the potential role of pleiotropy in intra-floral modularity is transgenic perturbation of candidate genes that might affect multiple floral traits within a module while affecting other traits or modules far less. In this study, we employ this transgenic approach to test modular regulation of the lengths of corolla tube, stamen, and pistil, in the hummingbird-pollinated monkeyflower species *M. verbenaceus*. *M. verbenaceus* is part of the *Mimulus lewisii* species complex, a powerful new model for developmental genetics and evo-devo studies of pollinator-associated floral traits [25]. We chose this species because it has a small stature but large, showy flowers (Fig. 1), a short generation time, and relatively small genome (~500 MB) with high-quality genomic resources (http://mimubase.org/), and most importantly, is readily amenable to *Agrobacterium*-mediated stable transformation [26].

Choosing the appropriate candidate genes, however, was not trivial. Although a large number of genes are known to control plant organ size in *Arabidopsis* [27–29], few genes are known to specifically control floral organ length with little effect on other parts of the plant, especially in sympetalous species. Given that plant organ elongation often involves coordinated actions of gibberellin (GA), brassinosteroid (BR), and auxin, we reasoned that an “integrator” of these hormone signaling pathways might be a good candidate. In particular, if this integrator gene has multiple functionally redundant paralogs in the genome, one of these gene family members could be preferentially expressed in floral organs, thereby regulating the elongation of multiple floral organs with minimal effect on vegetative tissues. The *PACLOBUTRAZOL RESISTANCE* (*PRE*) gene family fits these criteria. There are several closely related *PRE* paralogs in most plant genomes [30–32]. They encode small helix–loop–helix (HLH) proteins (90 ~ 110 amino acids) that act downstream of and integrate GA, BR, and auxin signaling to promote cell elongation in *Arabidopsis* [30, 33–35], tomato (*Solanum lycopersicum*) [36], cotton (*Gossypium spp.*) [32], and rice (*Oryza sativa*) [31, 35, 37]. As such, we considered them promising candidates in the modular regulation of floral organ elongation.

Here we report the characterization of *PRE* homologs in *M. verbenaceus* and transgenic perturbation of *MvPRE1*, the only homolog preferentially expressed in floral organs. Our results demonstrate that *MvPRE1* positively regulates corolla tube length, petal lobe length, stamen length, and pistil length, but not calyx or leaf length. We suggest that genes like *MvPRE1* may occupy a specialized hub position [38] in a genetic network and regulate multiple traits in a functional module while having little effect on other modules, and thus provide the suitable genetic materials for the evolution of phenotypic integration and modularity through pleiotropy.

## Results

### *MvPRE1* is the major *PRE*-like gene expressed during flower development in *M. verbenaceus*

**Figure 2:**
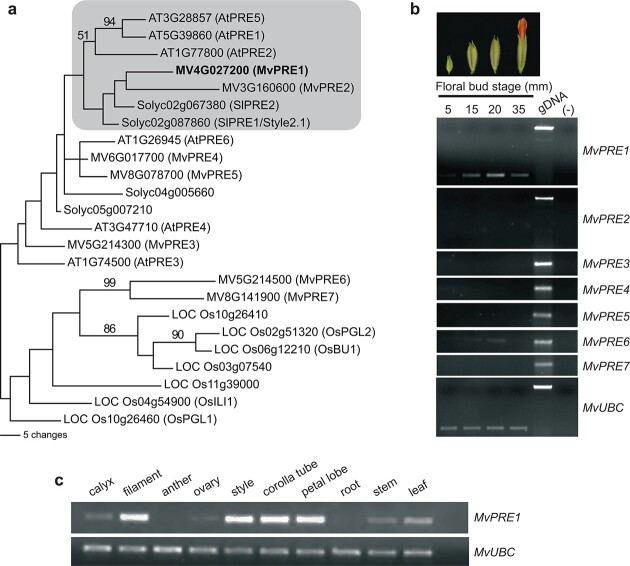
Expression analysis of *PRE1*-like genes in *Mimulus verbenaceus*. (a) Phylogenetic tree based on parsimony analysis, showing relationships of PRE1-like proteins in *M. verbenaceus*, *Arabidopsis*, tomato, and rice. Bootstrap support values >50% are indicated along the branches. The maximum likelihood tree (Supplementary Fig. S1) shows a very similar topology. (b) RT-PCR of *MvPRE* genes (28 cycles) at four different floral bud stages in MvBL. Genomic DNA (gDNA) was used as positive control for primer quality and water was used as negative control. *MvUBC* (27 cycles) was used as a reference gene. (c) RT-PCR of *MvPRE1* (28 cycles) in root, stem, leaf, and various floral tissues dissected from 20-mm floral buds. Root, stem, and leaf tissues were harvested from 5-week old seedlings. *MvUBC* (28 cycles) was used as a reference gene.

We identified seven *PRE*-like genes, *MvPRE1-MvPRE7*, from the MvBL genome by TBLASTN searches. They encode proteins that are 91 ~ 105 a.a. long and share sequence similarity with AtPRE1 across the entire length (Fig. 2a; Supplementary File S1). To determine which *MvPRE* genes may function in flower development, we performed RT-PCR across four stages of floral bud development (i.e. 5-mm, 15-mm, 20-mm, and 35-mm), and found that *MvPRE1* is the only *PRE*-like gene with readily detectable expression across multiple floral developmental stages, with peak expression level at the 20-mm stage (Fig. 2b) when the corolla tube is rapidly growing through cell elongation [39]. Phylogenetic analysis suggests that *MvPRE1* is closely related to tomato *SlPRE1/Style2.1* and *SlPRE2* (Fig. 2a; Supplementary Fig. S1). We further examined the spatial expression pattern of *MvPRE1* in vegetative tissues and various floral organs dissected from 20-mm floral buds, and observed that *MvPRE1* is highly expressed in the corolla tube, petal lobe, filament, and style, with intermediate expression levels in the leaf and stem, weak expression in the calyx and ovary, and no detectable expression in the anther and root (Fig. 2c).

### *MvPRE1* knockdown causes coordinated decreases in the lengths of corolla tube, style, and stamen

**Figure 3:**
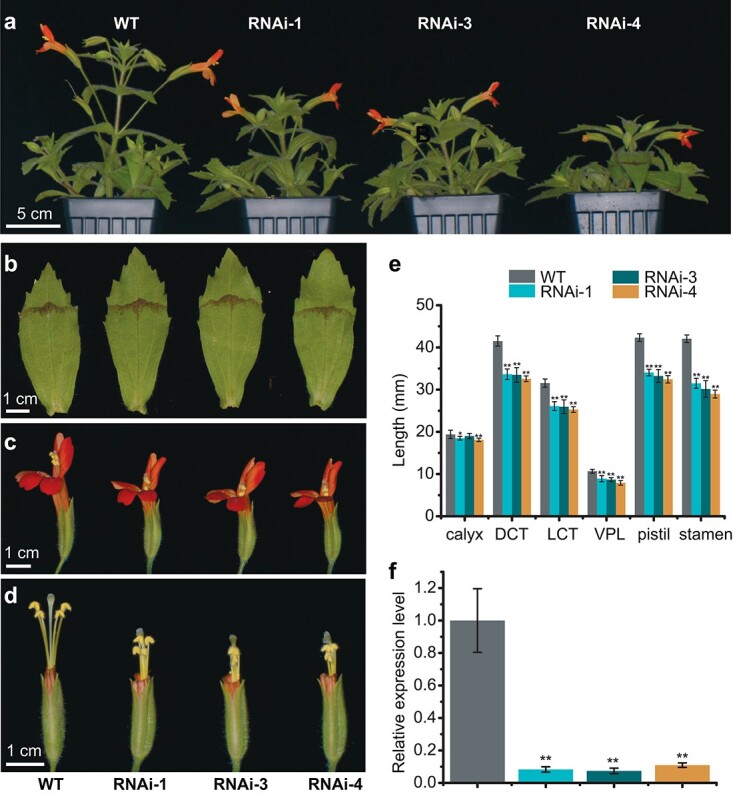
Phenotypes of *MvPRE1* RNAi transgenics. (a) Side view of the whole plant of wild type (WT) and three strong RNAi lines. The WT, RNAi-1, and RNAi-3 plants were 11-week old; the RNAi-4 plant was 10-week old. (b) The largest leaf (the 4th or 5th node on the main stem of 12-week old plants). (c and d) Side view of the whole flowers (c) or flowers with part of the corolla removed to expose the stamens and stigmas (d). Genotypes in (b-d) are shown in the same order as in (a). (e) Length measurements (n = 10 for each genotype). DCT: dorsal corolla tube; LCT: lateral corolla tube; VPL: ventral petal lobe. Error bars are 1 SD. (f) Relative transcript level of *MvPRE1* in 20-mm whole floral buds, as determined by RT-qPCR. Error bars are 1SD from three biological replicates. Asterisks indicate differences from the wild type (WT) (^*^ p^ ^< 0.05, ^**^ p < 0.01, student’s t test).

The spatial pattern of *MvPRE1* expression suggests that it may play an important role in the elongation of the corolla, stamen filament, and style. To test this hypothesis, we knocked down the expression of *MvPRE1* by transforming MvBL with an RNAi plasmid. We obtained five stable transgenic RNAi lines, three of which showed strong phenotypes and the other two showed intermediate phenotypes. Compared to the wild type, the three strong RNAi lines (RNAi-1, RNAi-3, RNAi-4) exhibited a moderately shorter stature (Fig. 3a) and slightly longer leaves (Fig. 3b; Supplementary Fig. S2a), but no obvious change in other vegetative characters. Intriguingly, the three floral traits that often show strong genetic correlations in sympetalous species (i.e. corolla tube length, stamen length, and pistil length) exhibited coordinated changes in these RNAi lines: the corolla tube, stamen, and pistil are reduced in length by ~20%, 28%, and 21%, respectively, compared to the wild type (Fig. 3c-e). Petal lobe length was also reduced to a similar degree (~20%), but calyx length showed little change (Fig. 3e). These results are consistent with the tissue-specific expression pattern of *MvPRE1* in the wild-type flower, in that *MvPRE1* has relatively high expression levels in the corolla tube, petal lobe, stamen filament, and style, but low expression levels in the leaf and calyx (Fig. 2c). To verify that these phenotypes are indeed caused by the knockdown of *MvPRE1*, we performed RT-qPCR on whole floral buds at the 20-mm stage and found that the expression level of *MvPRE1* decreased nearly 10-fold in the strong RNAi lines (Fig. 3f). In addition, the different phenotypic strengths displayed by the intermediate and strong RNAi lines furnished an opportunity to test phenotypic correlation quantitatively (Supplementary Fig. S3). As expected, the corolla tube length, stamen length, and pistil length were tightly correlated with each other (Supplementary Fig. S3f). The internode length was also correlated with the three floral traits, but less strongly than that among the three floral traits. By contrast, leaf length showed only weak correlation with internode length, and no correlation with any of the three floral traits (Supplementary Fig. S3f). Taken together, these results suggest that *MvPRE1* coordinately regulates the length of multiple pollinator-associated floral traits (i.e. corolla tube, petal lobe, stamen, and style), but plays little role in calyx or leaf elongation in *M. verbenaceus*.

### *MvPRE1* overexpression leads to increases in corolla tube and stamen lengths, but not pistil length

**Figure 4:**
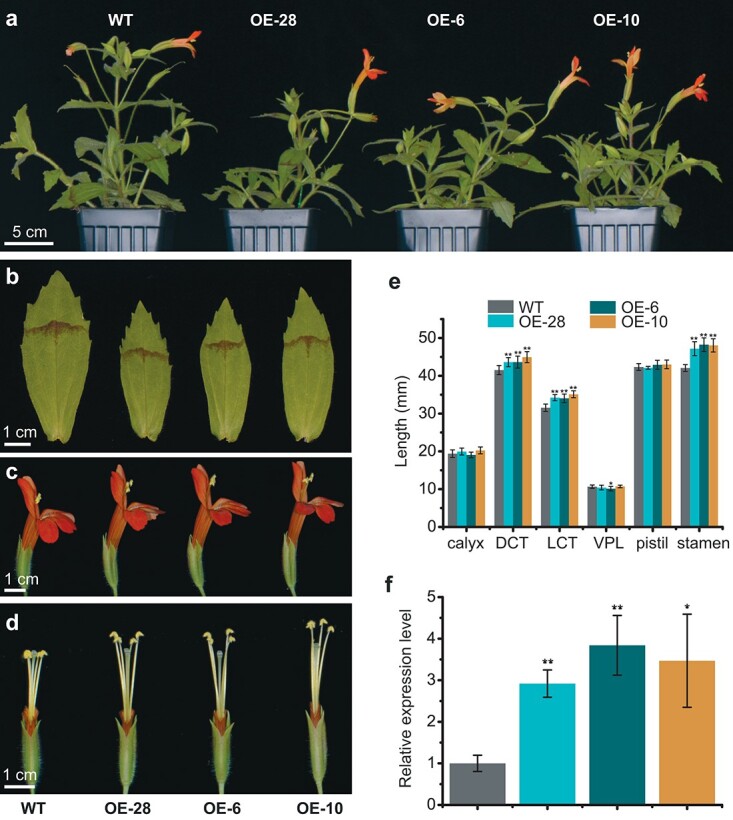
Phenotypes of *MvPRE1* overexpression transgenics. (a) Side view of 11-week old whole plants of the wild type (WT) and three strong overexpression lines. (b) The largest leaf (the 4th or 5th node on the main stem of 12-week old plants). (c and d) Side view of the whole flowers (c) or flowers with part of the corolla removed to expose the stamens and stigmas (d). Genotypes in (b-d) are shown in the same order as in (a). (e) Length measurements (n = 10 for each genotype). DCT: dorsal corolla tube; LCT: lateral corolla tube; VPL: ventral petal lobe. Error bars are 1 SD. (f) Relative transcript level of *MvPRE1* in 20-mm whole floral buds, as determined by RT-qPCR. Error bars are 1SD from three biological replicates. Asterisks indicate differences from the wild type (WT) (^*^ p < 0.05, ^**^ p < 0.01, student’s t test).

Given that knockdown of *MvPRE1* in MvBL causes decreases in the lengths of the corolla tube, stamen, pistil, and petal lobe, we expected that overexpression of *MvPRE1* would increase the lengths of these floral organs, and maybe even increase the length of calyx, where the endogenous expression level of *MvPRE1* is low. To test these predictions, we transformed MvBL with a *35S:MvPRE1-YFP* plasmid. We obtained 40 stable transgenic lines, 11 of which showed obvious phenotypes and three (OE-28, OE-6, and OE-10) were selected for further analysis. As expected, corolla tube and stamen lengths increased significantly in the overexpression lines compared to the wild type (Fig. 4c-e). However, pistil length did not change (Fig. 4e). As a result, the anthers are clearly positioned above the stigma in the overexpression lines, whereas in the wild type the anthers are at similar height to the stigma (Fig. 4d). We did not observe any obvious change in petal lobe or calyx length either (Fig. 4e). The overexpression lines also showed changes in vegetative tissues: the whole plant appears less robust, and leaves are shorter and narrower than the wild type (Fig. 4a,b; Supplementary Fig. S2b). RT-qPCR experiments verified that the expression levels of *MvPRE1* increased 3 ~ 4-fold in the three selected overexpression lines (Fig. 4f). The *35S:MvPRE1-YFP* transgene also allowed us to examine subcellular localization of the MvPRE1 proteins, which are predominantly localized in the nucleus, with weaker YFP signals also detectable in the cytosol (Supplementary Fig. S4). This pattern of subcellular localization is very similar to AtPRE1 and AtPRE3/ATBS1 in *Arabidopsis* [30, 34]. Taken together, these results further demonstrate that *MvPRE1* positively regulates elongation of some floral organs, but its function is tissue dependent, as it appears to have no effect on the calyx and have negative effect on leaf size.

### *MvPRE1* promotes cell elongation in *M. verbenaceus* flowers

**Figure 5:**
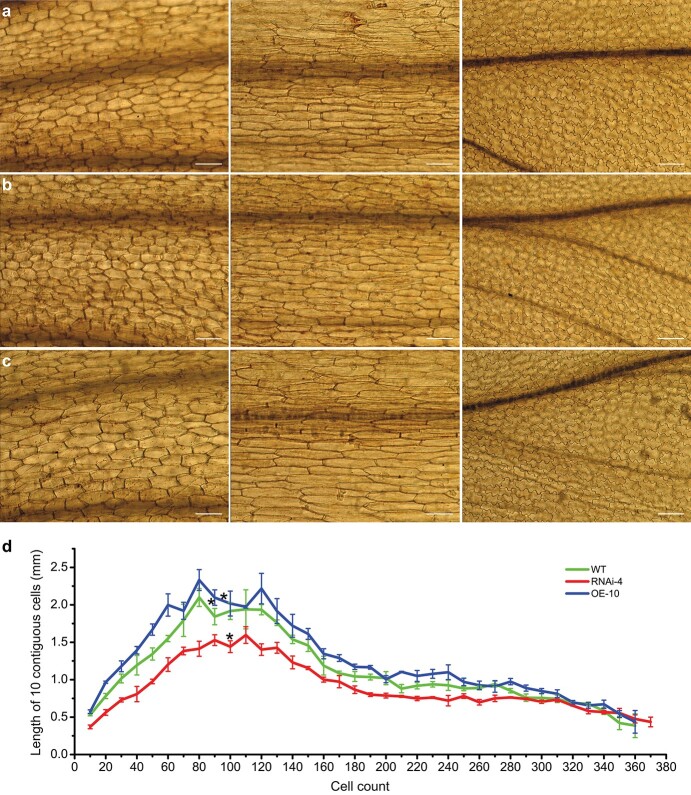
Cell profiles along the dorsal side of the corolla tubes in the wild type (a), RNAi-4 (b), and OE-10 (c). Individual images show the morphology of cells at the base of the corolla tube (left), near the stamen insertion site (center), and at the top of the corolla tube (right). Scale bars are 100 μm. (d) Cell number and length along the outer (abaxial) epidermis of the dorsal corolla tube. The asterisks indicated stamen insertion sites. Error bars are 1SE. *N* = 3 for each genotype.

To determine whether the floral organ length changes in the *MvPRE1* RNAi and overexpression lines are due to change in cell number or cell length, we profiled cell number and size along the dorsal side of the corolla tube of open flowers from the wild type (MvBL), RNAi line 4 (RNAi-4), and overexpression line 10 (OE-10). Consistent with a previous study [39], we found that in the wild-type MvBL, cell length increases gradually from the base of the corolla tube to the stamen insertion site, then plateaus for ~50 cells with maximal length before gradually decreasing towards the distal end (Fig. 5a,d). Accordingly, cell shape changes from more rounded at the base to elongated rectangular in the plateau zone to puzzle-shaped at the distal end (Fig. 5a). RNAi-4 and OE-10 showed the same pattern of cell length variation along the corolla tube and had similar cell numbers to the wild type (Fig. 5b-d). The main difference is that RNAi-4 has shorter cells than the wild type, especially in the zone near the stamen insertion site (from the 50th to the 150th cell), and OE-10 has longer cells (Fig. 5d; Supplementary Table S1). These results suggest that *MvPRE1* regulates floral organ length by promoting cell elongation instead of cell division, consistent with the known function of its homologs in *Arabidopsis*, rice, and cotton [32, 33, 35].

## Discussion

In this study, we have shown that knocking down the expression of a *PRE*-like gene in the hummingbird-pollinated monkeyflower species *M. verbenaceus* caused simultaneous decreases in the lengths of corolla tube, petal lobe, stamen, and pistil, but little change in calyx and leaf lengths or organ width (Fig. 3b-e; Supplementary Fig. S2a). For sympetalous species, the lengths of corolla tube, stamen, and pistil often show strong phenotypic and genetic correlation independent of other floral or vegetative traits, and these organs are considered to be a functional module [10–18]. Because the corolla tube can direct or limit pollinator entrance, integration of tube, stamen, and pistil lengths maintains the anthers and stigma in the appropriate position for contacting the pollinator [40, 41]. Moreover, coordination of the relative positions of the anthers and stigma (herkogamy) mediates the efficacy of pollen transfer [17, 42, 43]. Pleiotropy has long been implicated in such patterns of modular integration in sympetalous species [9, 44], but empirical evidence based on actual gene function is lacking. Our demonstration of the modular regulation of these traits by a single gene supports the role of pleiotropy in generating intra-floral integration.

The overexpression experiment suggests that the role of *MvPRE1* in organ elongation depends on the developmental context. While downregulation of *MvPRE1* affected all organs in the putative functional module, overexpression of *MvPRE1* in *M. verbenaceus* led to increased corolla tube and stamen length, as expected, but did not change pistil or calyx length (Fig. 4c-e). In the leaf, overexpression of *MvPRE1* even had the opposite effect: both leaf length and width were reduced (Fig. 4b; Supplementary Fig. S2b). Notably, *AtPRE1* overexpression in *Arabidopsis* also resulted in narrower leaves [30], indicating that the leaf phenotypes we observed are not an oddity unique to *M. verbenaceus*.

In *Arabidopsis*, AtPRE1 is part of a triantagonistic HLH/bHLH system that controls cell elongation. In this system, the bHLH ACTIVATOR FOR CELL ELONGATION (ACE) transcription factors bind to the promoters and directly activate *EXPANSIN* genes, leading to cell elongation. A HLH protein, IBH1, interacts with ACEs and interferes with the DNA-binding activities of ACEs, thereby negatively regulating cell elongation. PRE1 is the third HLH component and its interaction with IBH1 releases ACEs, restoring the transcriptional activities of ACEs [33, 45]. The balance of these three HLH/bHLH proteins is important to determine the ultimate cell length. Our cell number and length profiling (Fig. 5) demonstrated that MvPRE1 promotes cell elongation in *M. verbenaceus*, just as the PRE-like proteins in other plants. Assuming MvPRE1 also functions in a triantagonistic HLH/bHLH system, one possible explanation of the different effects of *MvPRE1* overexpression on the corolla tube, stamen, and pistil is the different amounts of MvIBH1 and MvACEs in these floral organs. If the endogenous levels of MvIBH1 are relatively high in the corolla tube and stamen, then providing more MvPRE1 by the transgene can release more MvACEs from MvIBH1 and consequently produce longer cells in the corolla tube and stamens. By contrast, if MvIBH1 abundance is already low in the pistil, providing additional MvPRE1 will not make a difference. The lack of calyx phenotypes in either the *MvPRE1* RNAi or overexpression lines suggests that calyx length may be regulated by mechanisms independent of this HLH/bHLH system. The opposite effect of *MvPRE1* overexpression on the leaf is puzzling.

Regardless of the exact underlying mechanisms, the fact that transgenic manipulations of *MvPRE1* trigger correlated responses of the three floral organs (corolla, stamen, and pistil) that are distinct from the leaf or calyx is evolutionarily and functionally significant. An important criterion of modularity is that the characters within a module are tightly correlated with each other, but less strongly correlated with those outside of the module [1, 8, 22]. *MvPRE1* provides an example of how this can be realized by the pleiotropic effect of a single gene. Although manipulation of *MvPRE1* did not exclusively affect the organs of the putative functional module, the effects on the serially homologous calyx and vegetative leaves were far less (Fig. 3; Supplementary Fig. S3).

Pleiotropy and modularity may have played a critical role in the evolutionary diversification of organismal forms in general [21, 22, 24] and flower form and function in particular [9, 44], because selection on one trait will result in correlated changes in functionally related traits. Indeed, floral traits associated with pollinator shifts have been subjected to quantitative trait locus (QTL) analysis in a wide range of sympetalous taxa, including *Mimulus* [11, 46], *Leptosiphon* [47]*, Ipomopsis* [48], *Penstemon* [18], *Petunia* [49], and *Ipomoea* [50]. These studies showed that corolla tube, stamen, and style lengths are not only genetically correlated but also frequently co-localized to the same QTL, consistent with a role of pleiotropic QTLs underlying intra-floral integration and in facilitating rapid floral form diversification.

It should be noted that our results on *MvPRE1* only show that pleiotropy *can* produce intra-floral integration, it does not show pleiotropy actually *did* cause intra-floral integration during *Mimulus* evolution. To demonstrate the latter, one would need to first identify the presumptive gene that causes intra- or inter-specific covariation of traits that constitute a functional module, then demonstrate the phenotypic consequence and pleiotropic effect of the causal gene by generating high-resolution introgression lines (i.e. allelic substitution at single-gene level between the two forms) or direct transgenic manipulations. Unfortunately, although efforts in identifying causal genes responsible for intra- or inter-specific flower color variation in *Mimulus* were successful [51–56], progress in genetic mapping of floral organ length variation has been slow, partly because organ length tends to be more polygenic than pigmentation traits [11, 46, 57]. However, with chromosome-level genome assemblies for multiple *Mimulus* species becoming available recently (http://mimubase.org/), identification of casual genes underlying floral organ length variation should be soon within reach.

This study also raises the question of how many other genes may behave like *MvPRE1* in modular regulation of floral traits. The majority of genes reported to control floral organ size in *Arabidopsis* also tend to have strong pleiotropic effects in vegetative tissues [27–29]. We still know very little about which genes specifically control floral organ size, shape, number, and spatial arrangement without affecting other plant parts, especially in non-*Arabidopsis* species. However, it is not difficult to envision the general properties of such genes: (i) They are likely “hub” genes in a gene regulatory network to control the development of multiple traits [23]; (ii) They should have closely related paralogs in a genome to allow parcellation (e.g. through sub-functionalization or evolution of tissue specific expression patterns) [22]. Given the impressive pace of technological improvements (e.g. long-read sequencing, genome editing) in the past few years, both classical model systems for floral organ elaboration like snapdragon [58] or petunia [59] and more recently developed plant systems such as *Aquilegia* [60], *Sinningia* [61], *Torenia* [62], *Mimulus* [25], and *Nigella* [63] are becoming more and more accessible to detailed genetic and developmental analyses. We are optimistic that studies in these systems will reveal the genetic networks that can regulate multiple floral traits in a functional module but meanwhile have little effect on other modules, and that investigations on the role of pleiotropy in the evolution of floral integration and intra-floral modularity will begin to focus on specific genes instead of presumptive “loci” without any knowledge of their molecular identities.

## Materials and methods

### Plant materials and growth conditions

The *M. verbenaceus* inbred line MvBL (wild type) was previously described in [26]. All plants were grown in the University of Connecticut research greenhouses under natural light supplemented with sodium vapor lamps, ensuring a 16-hr day length with a light intensity of 110–160 μmol·m^−2^·s^−1^. Plants were fertilized 2–3 timesper week.

### Sequence retrieval and phylogenetic analysis

The amino acid (a.a.) sequence of *Arabidopsis* AtPRE1 (AT5G39860, 92 a.a.) was used as query to retrieve the *M. verbenaceus* homologs from the MvBL genome (version 2.0; http://mimubase.org/). TBLASTN searches, as implemented in the program TARGeT (http://new-cizin.cyverse.org/) [64], resulted in seven genes encoding proteins that are 91–105 a.a. long and share sequence similarity with AtPRE1 across the entire length. We named these genes *MvPRE1-MvPRE7*. Multiple sequence alignment (Supplementary File S1) of all PRE-like proteins from *Arabidopsis*, tomato, rice, and *M. verbenaceus* were performed in AliView [65]. Phylogenetic analyses of the amino acid sequence alignment were performed using both the parsimony criterion and the maximum likelihood method. Parsimony analysis was performed using PAUP^*^ version 4.0a168 [66], with 200 random stepwise addition replicates and TBR branch swapping with MULTREES option effective. Clade support was determined by bootstrap analyses of 200 replicates. Maximum likelihood analysis was performed using MEGA X [67] with the JTT amino acid substitution model and 1000 bootstrap replicates.

### Expression analysis using RT-(q)PCR

RNA extraction and cDNA synthesis were as described in [53]. Briefly, total RNA was extracted using the Spectrum Plant Total RNA Kit (Sigma-Aldrich). cDNA was synthesized from 1 μg of the DNaseI (Invitrogen) treated RNA using the SuperScript III First-Strand Synthesis System (Invitrogen) or GoScript™ Reverse Transcription Mix (Promega), then diluted 10-fold before RT-(q)PCR. RT-PCR was performed to determine the expression patterns of all seven *MvPRE* genes in different floral developmental stages of MvBL and the expression pattern of *MvPRE1* in various tissue types. The *M. verbenaceus* ortholog of *Arabidopsis* ubiquitin-conjugating enzyme gene (At5g25760), *MvUBC*, was used as a reference gene following [53]. Whole floral buds at 20-mm stage were used for assaying relative transcript levels of *MvPRE1* in wild-type MvBL and transgenic lines by RT-qPCR, which was performed using iQ SYBR Green Supermix (Bio-Rad) and a CFX96 Touch Real-Time PCR Detection System (Bio-Rad). For RT-qPCR experiments, three biological replicates were included for each genotype. Each replicate was a distinct 20-mm floral bud. The cDNA samples were amplified for 40 cycles of 95°C for 15 s and 60°C for 20 s. Amplification efficiencies for each primer pair were calculated using critical threshold values obtained from a dilution series (1:4:1:8:1:16:1:32) of pooled cDNAs. Relative expression of *MvPRE1* compared to the reference gene (*MvUBC*) was estimated using the formula (E_ref_) ^CP (ref)^/(E_target_) ^CP (target)^. The seven *MvPRE* paralogs are so divergent from each other at the nucleotide level (Supplementary Figure S5) that designing paralog-specific primers was straightforward. To further verify the specificity of primers, each primer was BLASTed against the MvBL genome in the TARGeT platform (http://new-cizin.cyverse.org/) [64] with an E-value cut-off of 0.1, ensuring that each primer has only one match in the genome. The primers used for RT-(q) PCR were listed in Supplementary Table S2.

### Plasmid construction and plant transformation

An RNA interference (RNAi) plasmid was constructed to knock down the expression of *MvPRE1* in MvBL. Since the *MvPRE1* gene is quite small, and BLASTing the full-length coding sequence (CDS) (282 bp, without the stop codon) against the MvBL genome found no other genomic regions that perfectly match this CDS for a contiguous block longer than 16 bp (i.e. the *MvPRE1* sequence is highly specific at the nucleotide level), we used the entire CDS for the RNAi plasmid. To this end, we first amplified the CDS from MvBL cDNA with the Phusion High-Fidelity DNA Polymerase (New England Biolabs), cloned it into the pDONOR207 vector (Invitrogen) by BP cloning reaction, and then cloned it into the pB7GWIWG2(I) vector [68] in both sense and antisense directions by LR recombination.

To build the overexpression plasmid, the full-length *MvPRE1* CDS (without the stop codon) was first cloned into the pDONOR207 vector (Invitrogen) by BP reaction, and then cloned into the destination vector pEarleyGate 101 [69] by LR recombination. The pEarleyGate 101 vector drives transgene expression by the cauliflower mosaic virus (CaMV) 35S promoter and contains the YFP CDS fused in frame with the 3′ end of the gene of interest (*MvPRE1*).

After verifying the plasmid sequence by Sanger sequencing, each final plasmid was transformed into *Agrobacterum tumefaciens* strain GV3101 and then transformed into the plants following the “floral spray/vacuum infiltration” protocol described in [53]. The primers used for plasmid construction were listed in Supplementary Table S2.

### Phenotypic analysis

To quantify the phenotypic differences among wild type, *MvPRE1* RNAi, and *MvPRE1* overexpression lines, we measured several floral traits using digital calipers, including lateral corolla tube (LCT) length, dorsal corolla tube (DCT) length, ventral petal lobe (VPL) length and width (Fig. 1b,c), pistil length (from the receptacle to the stigma), stamen length (from the receptacle to the anther), and calyx length. Ten fully open flowers were measured for each genotype. We also measured the length and width of the largest leaves (usually the fourth pair on the main stem) of mature plants. To determine whether the phenotypic differences between the wild type and the transgenic lines are due to cell number or cell size, we profiled the cell number and cell length along the dorsal side of the corolla tube (Fig. 1c) of mature flowers using a compound light microscope, following [39].

## Acknowledgements

We thank Matt Opel and Meghan Moriarty for plant care in the UConn EEB Research Glasshouses, Dr. Mei Liang for assistance in confocal imaging, and Vandana Gurung for assistance in cell measurements. This work was supported by National Science Foundation grants IOS-1755373 and IOS-1827645 to Y-W.Y. H.C. was partially supported by a fellowship from China Scholarship Council.

## Author contributions

All authors designed the research; H.C., Z.X., and B.D. performed the experiments; H.C. and Y-W.Y. analyzed the data; H.C., P.K.D., and Y-W.Y. wrote the manuscript with input from all authors.

## Data Availability

The sequence data that supports the findings of this study are available in Mimubase at http://mimubase.org/ with the following accession numbers: MvPRE1 (MV4G027200), MvPRE2 (MV3G160600), MvPRE3 (MV5G214300), MvPRE4 (MV6G017700), MvPRE5 (MV8G078700), MvPRE6 (MV5G214500), MvPRE7 (MV8G141900).

## Conflict of interest

The authors declare no conflict of interest.

## Supplementary data

Supplementary data is available at *Horticulture Research* online.

## Supplementary Material

Web_Material_uhac168
