# Supplementary material for: Modular regulation of floral traits by a PRE1 homolog in Mimulus verbenaceus: implications for the role of pleiotropy in floral integration
Source: Hortic Res. 2022 Jul 27;9:uhac168. doi: 10.1093/hr/uhac168 (PMC9531339; doi:10.1093/hr/uhac168)
Supplement: Web_Material_uhac168 [file web_material_uhac168.zip › Figure S1.docx]

**
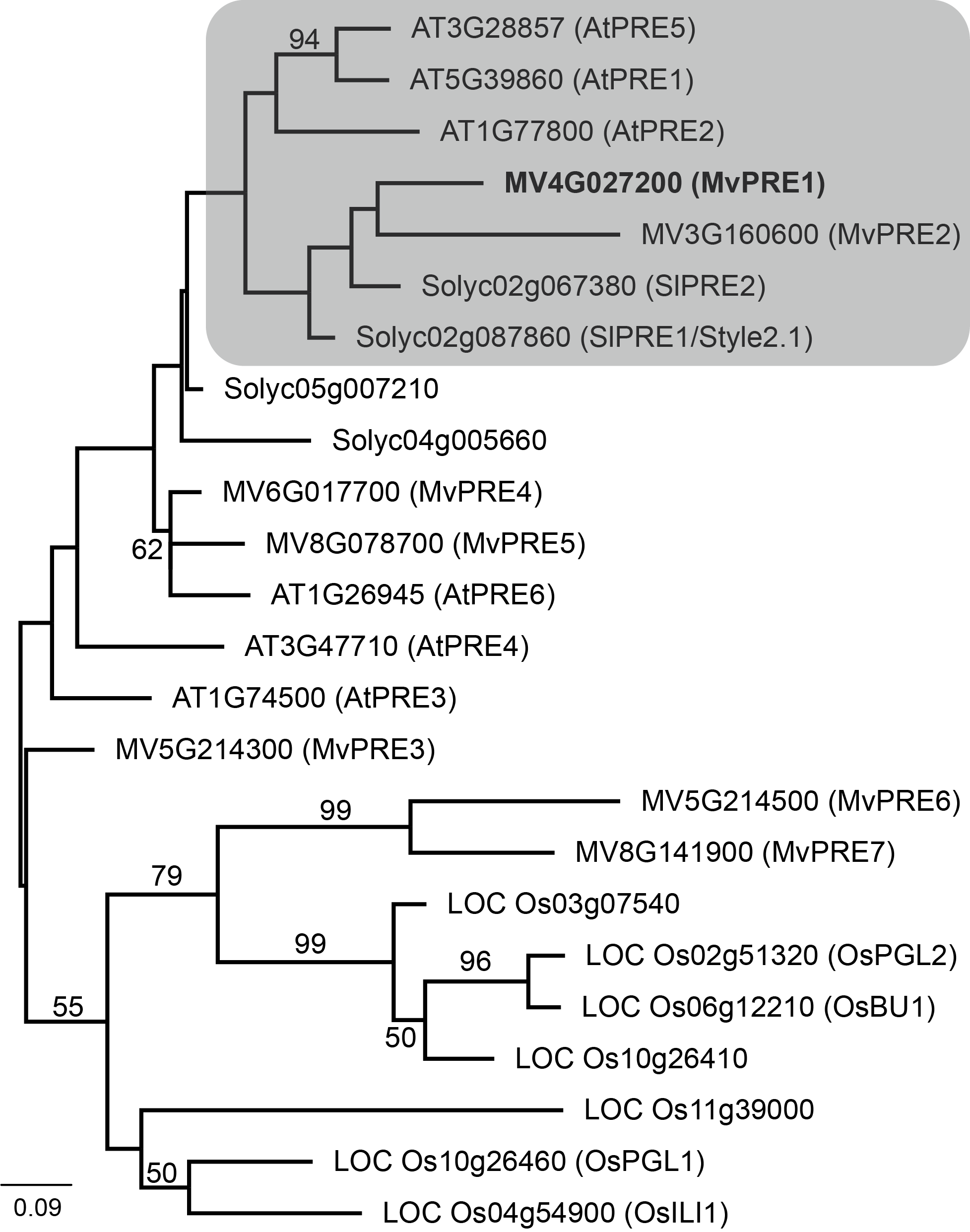
**

**Fig. S1** Maximum likelihood tree constructed using MEGA X, showing relationships of PRE1-like proteins in *M. verbenaceus*, *Arabidopsis*, tomato, and rice. Bootstrap support values >50% are indicated along the branches.
