# Supplementary material for: Modular regulation of floral traits by a PRE1 homolog in Mimulus verbenaceus: implications for the role of pleiotropy in floral integration
Source: Hortic Res. 2022 Jul 27;9:uhac168. doi: 10.1093/hr/uhac168 (PMC9531339; doi:10.1093/hr/uhac168)
Supplement: Web_Material_uhac168 [file web_material_uhac168.zip › Figure S2.docx]

**
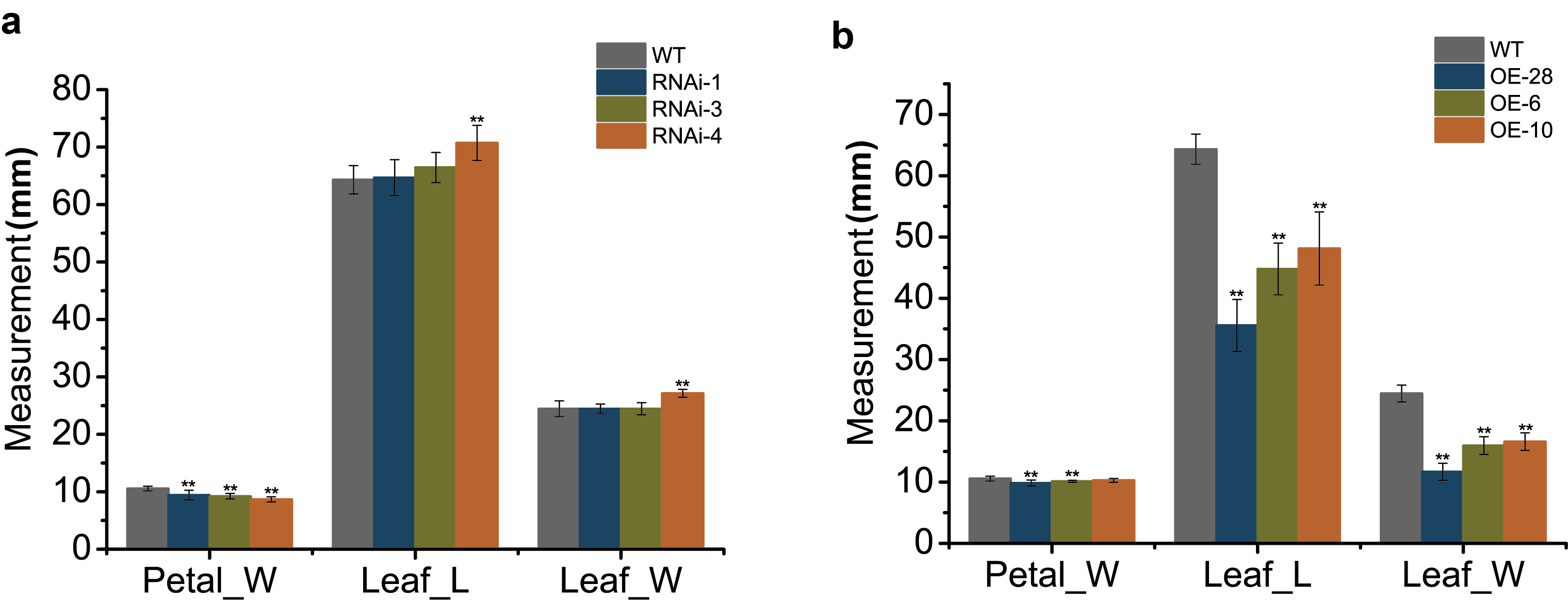
**

**Fig S2.** Measurements of ventral petal lobe width (n = 10 for each genotype), leaf length (n = 6), and leaf width (n = 6) in three strong *MvPRE1* RNAi lines (a) and three strong overexpression lines (b).
