# Supplementary material for: Modular regulation of floral traits by a PRE1 homolog in Mimulus verbenaceus: implications for the role of pleiotropy in floral integration
Source: Hortic Res. 2022 Jul 27;9:uhac168. doi: 10.1093/hr/uhac168 (PMC9531339; doi:10.1093/hr/uhac168)
Supplement: Web_Material_uhac168 [file web_material_uhac168.zip › Figure S3.docx]

**
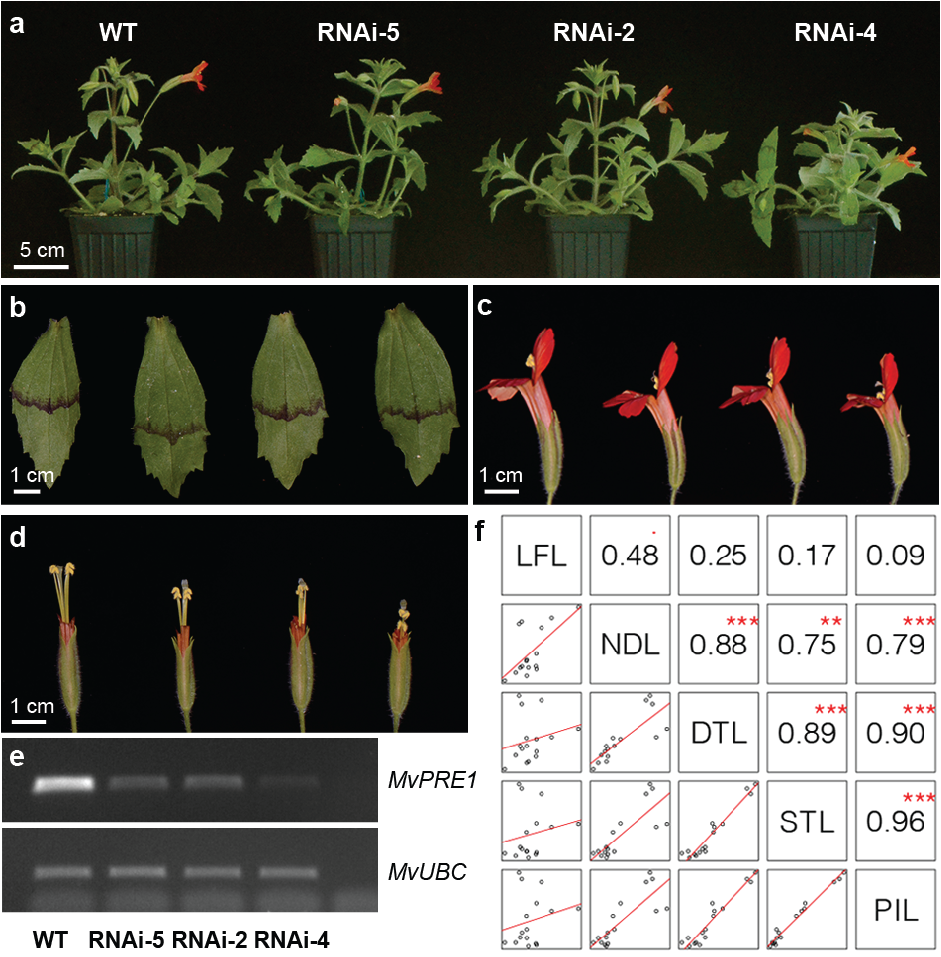
**

**Fig S3.** Phenotypic correlation in *MvPRE1* RNAi lines. (a) Side view of the whole plant of wild type (WT), two RNAi lines (RNAi-5 and RNAi-3) with intermediate phenotypes, and one RNAi line (RNAi-4) with strong phenotypes. The plants were 10-week old. (b) The largest leaf (the 4th or 5th node on the main stem of 10-week old plants). (c) Side view of the whole flowers. (d) Side view of the flowers with part of the corolla removed to expose the stamens and stigmas. Genotypes in (b-d) are shown in the same order as in (a). (e) Relative transcript level of *MvPRE1* in 20-mm whole floral buds, as determined by RT-PCR; (f) Pairwise correlations among five traits measured from 16 plants including 3 individuals of RNAi-1, 3 individuals of RNAi-2, 3 individuals of RNAi-3, 2 individuals of RNAi-4, 2 individuals of RNAi-5, and 3 individuals of wild-type MvBL. Spearman’s correlation coefficients (r) are provided above the diagonal, which were calculated using the function *cor* in R v. 3.6.0. Scatterplots are provided below the diagonal. The asterisks indicate the significance levels of the correlations (**P < 0.01, ***P < 0.001), which were calculated by the function *cor.test* in R v. 3.6.0. The correlation results were visualized using function *pairs* in R v. 3.6.0. LFL: leaf length (the 4th or 5th node on the main stem of 11-week old plants); NDL: internode length (between the 3rd and 5th nodes of 11-week old plants); DTL: dorsal corolla tube length; STL: stamen length; PIL: pistil length.
