# Supplementary material for: Modular regulation of floral traits by a PRE1 homolog in Mimulus verbenaceus: implications for the role of pleiotropy in floral integration
Source: Hortic Res. 2022 Jul 27;9:uhac168. doi: 10.1093/hr/uhac168 (PMC9531339; doi:10.1093/hr/uhac168)
Supplement: Web_Material_uhac168 [file web_material_uhac168.zip › Figure S5.docx]

**
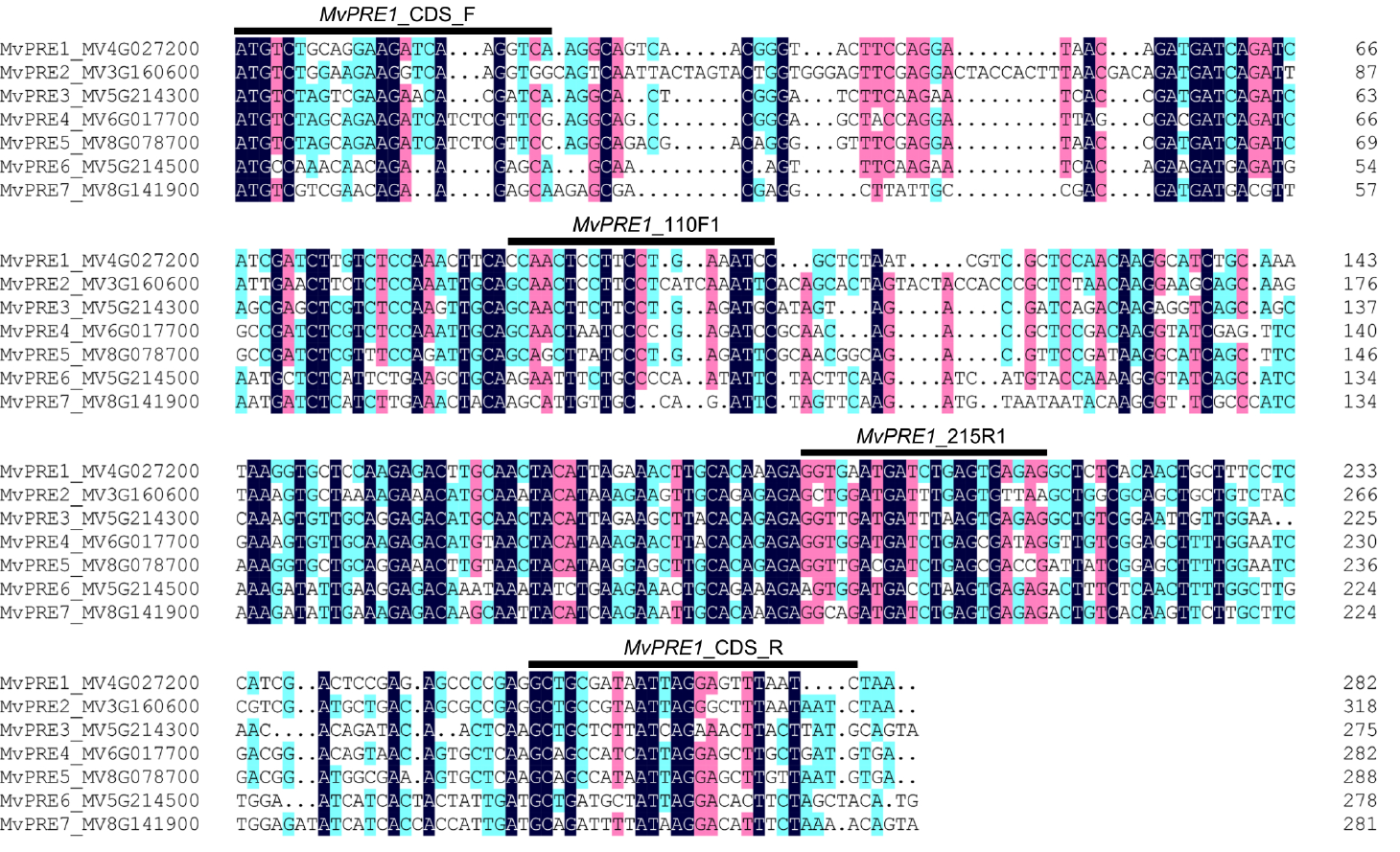
**

**Fig S5.** Alignment of the coding DNAs of the seven *MvPRE* paralogs. Sequence similarity levels are indicated by shades of black (100%), red (75-99%), and cyan (50-74%).
