# Supplementary material for: Modular regulation of floral traits by a PRE1 homolog in Mimulus verbenaceus: implications for the role of pleiotropy in floral integration
Source: Hortic Res. 2022 Jul 27;9:uhac168. doi: 10.1093/hr/uhac168 (PMC9531339; doi:10.1093/hr/uhac168)
Supplement: Web_Material_uhac168 [file web_material_uhac168.zip › Supplementary_tables.docx]

**Table S1**. Average cell length (μm, mean ± SE) of every 10 contiguous cells along the dorsal side of the corolla tube (n = 3 mature flowers for each genotype).

| **Cell coordinate** | **WT** | **RNAi-4** | **OE-28** |
| --- | --- | --- | --- |
| 1-10 | 530.14±13.1 | 369.06±24.87 | 573.10±23.78 |
| 11-20 | 781.66±33.85 | 561.97±37.42 | 966.50±14.12 |
| 21-30 | 1016.13±59 | 728.69±27.04 | 1178.85±72.14 |
| 31-40 | 1192.97±130.31 | 807.60±102.07 | 1392.89±50.08 |
| 41-50 | 1343.13±81.58 | 976.25±30.2 | 1669.58±75.28 |
| 51-60 | 1549.55±41.36 | 1202.15±90.61 | 1997.83±149.08 |
| 61-70 | 1792.99±214.89 | 1383.07±50.72 | 1920.31±113.9 |
| 71-80 | 2098.12±119.31 | 1413.19±100.64 | 2331.49±139.12 |
| 81-90 | 1842.75±109 | 1528.02±71.87 | 2096.81±103.28 |
| 91-100 | 1917.15±113.23 | 1441.17±78.78 | 2016.76±166.57 |
| 101-110 | 1939.84±261.07 | 1596.35±111.37 | 1973.55±17.64 |
| 111-120 | 1934.22±68 | 1402.20±76.68 | 2217.18±203.87 |
| 121-130 | 1760.74±37.86 | 1426.12±72.18 | 1915.74±166.66 |
| 131-140 | 1538.15±56.14 | 1230.39±85.2 | 1720.10±153.56 |
| 141-150 | 1456.96±80.22 | 1155.79±20.55 | 1611.88±71.64 |
| 151-160 | 1187.25±125.35 | 1000.80±32.98 | 1346.04±67.23 |
| 161-170 | 1085.7±28.61 | 973.09±80.89 | 1288.69±44.64 |
| 171-180 | 1042.56±66.42 | 866.28±50.65 | 1171.69±36.15 |
| 181-190 | 1035.31±39.12 | 800.16±24.36 | 1165.35±24.81 |
| 191-200 | 1020.14±86.95 | 787.34±31.83 | 1006.25±47.46 |
| 201-210 | 880.61±55.67 | 776.84±12.98 | 1104.28±7.39 |
| 211-220 | 918.8±42.01 | 748.54±28.6 | 1049.95±72.64 |
| 221-230 | 938.08±35.99 | 761.74±12.28 | 1078.02±59.39 |
| 231-240 | 925.24±68.81 | 719.45±69.75 | 1100.03±98.39 |
| 241-250 | 882.37±29.77 | 780.77±30.59 | 970.13±49.74 |
| 251-260 | 888.94±25.7 | 698.42±36.96 | 925.27±55.39 |
| 261-270 | 942.01±38.46 | 750.41±42.82 | 909.71±77.29 |
| 271-280 | 852.94±29.01 | 762.49±10.04 | 974.10±42.04 |
| 281-290 | 759.45±49.83 | 743.90±13.56 | 887.72±40.51 |
| 291-300 | 755.73±62.6 | 705.41±22.22 | 847.64±34.44 |
| 301-310 | 743.76±49.66 | 733.29±27.91 | 811.47±53.46 |
| 311-320 | 688.51±27.41 | 652.28±4.52 | 697.95±23.87 |
| 321-330 | 679.5±20.43 | 581.63±37.37 | 656.70±38.52 |
| 331-340 | 581.82±46.61 | 566.78±21.83 | 671.93±54.91 |
| 341-350 | 421.91±89.66 | 554.86±61.06 | 541.99±45.31 |
| 351-360 | 385.77±159.25 | 475.53±54.35 | 436.69±151.27 |
| 361-370 | NA | 435.29±64.38 | NA |

**Table S2** Sequences of primers used in this study. The sequences highlighted in red are necessary for BP cloning. Note that the same *MvPRE1*_CDS_F & R primers were used for building the RNAi and overexpression plasmids.

| **Primer** | **Sequence (5’-3’)** | **Usage** |
| --- | --- | --- |
| *MvPRE1*_110F1 | CCAACTCCTTCCTGAAATCC | RT-(q)PCR |
| *MvPRE1*_215R1 | CTCTCACTCAGATCATTCACC | RT-(q)PCR |
| *MvPRE2*_105F1 | AAATTGCAGCAACTCCTTCC | RT-PCR |
| *MvPRE2*_185R1 | GCACTTTACTTGCTGCTTCC | RT-PCR |
| *MvPRE3*_185F1 | AGGTTGATGATTTAAGTGAGAGG | RT-PCR |
| *MvPRE3*_270R1 | GCATAAGTAAGTTTCTGATAAGAGC | RT-PCR |
| *MvPRE4*_190F1 | TGGATGATCTGAGCGATAGG | RT-PCR |
| *MvPRE4*_280R1 | CAGCAAGCTCCTAATGATGG | RT-PCR |
| *MvPRE5*_720F1 | CTGGAGAAGCAGTATCTTTGG | RT-PCR |
| *MvPRE5*_820R1 | TGATGAGACTCGTCGAAGG | RT-PCR |
| *MvPRE6*_120F1 | TCAGCATCAAAGATATTGAAGG | RT-PCR |
| *MvPRE6*_240R1 | TAGTAGTGATGATTCCACAAGC | RT-PCR |
| *MvPRE7*_160F1 | ATCAAGAAATTGCACAAAGAGG | RT-PCR |
| *MvPRE7*_240R1 | TGGTGATGATATCTCCAGAAGC | RT-PCR |
| *MIUBC*_F | GGCTTGGACTCTGCAGTCTGT | RT-(q)PCR |
| *MIUBC*_R | TCTTCGGCATGGCAGCAAGTC | RT-(q)PCR |
| *MvPRE1*_CDS_F | GGGGACAAGTTTGTACAAAAAAGCAGGCTTCATGTCTGCAGGAAGATCAAGGTCA | Plasmid construction |
| *MvPRE1*_CDS_R | GGGGACCACTTTGTACAAGAAAGCTGGGTCGATTAAACTCCTAATTATCGCAGC | Plasmid construction |
