# Supplementary figures and images for: Modular regulation of floral traits by a PRE1 homolog in Mimulus verbenaceus: implications for the role of pleiotropy in floral integration
Source: Hortic Res. 2022 Jul 27;9:uhac168. doi: 10.1093/hr/uhac168 (PMC9531339; doi:10.1093/hr/uhac168)

**
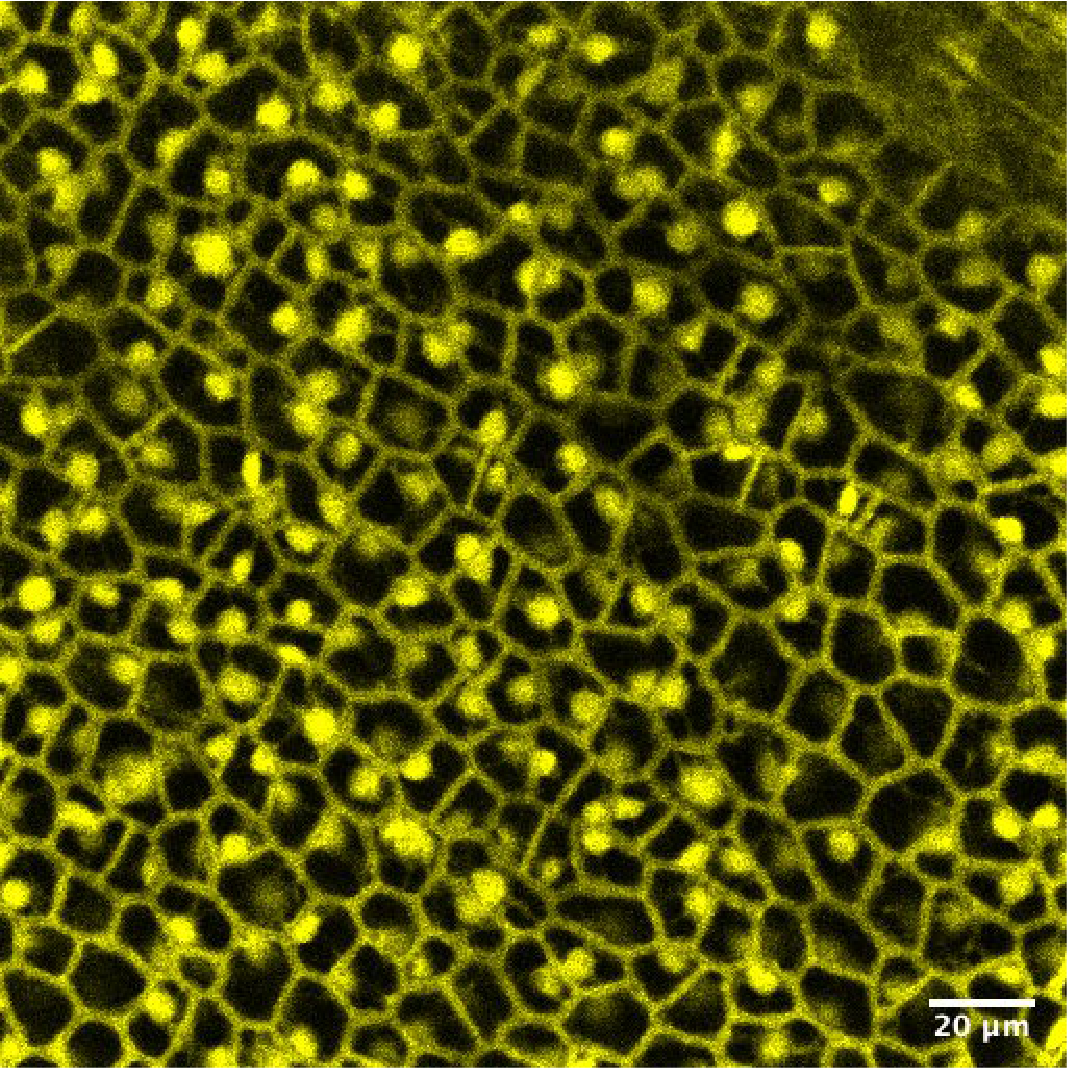
**

**Fig S4.** Subcellular localization of MvPRE1-YFP. Petal lobe of OE-28 at the 5-mm corolla stage.

Supplement: Web_Material_uhac168 [file web_material_uhac168.zip › Figure S4.docx]
